# Informing evidence-based policy during the COVID-19 pandemic and recovery period: learning from a national evidence centre

**DOI:** 10.1186/s41256-024-00354-1

**Published:** 2024-05-31

**Authors:** Alison Cooper, Ruth Lewis, Micaela Gal, Natalie Joseph-Williams, Jane Greenwell, Angela Watkins, Alexandra Strong, Denitza Williams, Elizabeth Doe, Rebecca-Jane Law, Adrian Edwards

**Affiliations:** 1 Wales Centre for Primary and Emergency Care Research (PRIME Centre Wales), Bangor, Wales; 2 Division of Population Medicine, Cardiff University, Cardiff, Wales; 3 North Wales Centre for Primary Care Research, Bangor University, Bangor, Wales; 4 Public Contributor, WCEC Public Partnership Group, Bangor, Wales; 5 Technical Advisory Cell, Welsh Government, Cardiff, Wales

**Keywords:** Evidence-based policy, Research prioritisation, Rapid evidence synthesis, Stakeholder engagement, Knowledge mobilisation

## Abstract

**Background:**

The COVID-19 pandemic demonstrated the vital need for research to inform policy decision-making and save lives. The Wales COVID-19 Evidence Centre (WCEC) was established in March 2021 and funded for two years, to make evidence about the impact of the pandemic and ongoing research priorities for Wales available and actionable to policy decision-makers, service leads and the public.

**Objectives:**

We describe the approaches we developed and our experiences, challenges and future vision.

**Program implementation:**

The centre operated with a core team, including a public partnership group, and six experienced research groups as collaborating partners. Our rapid evidence delivery process had five stages: 1. Stakeholder engagement (continued throughout all stages); 2. Research question prioritisation; 3. Bespoke rapid evidence review methodology in a phased approach; 4. Rapid primary research; and 5. Knowledge Mobilisation to ensure the evidence was available for decision-makers.

**Main achievements:**

Between March 2021–23 we engaged with 44 stakeholder groups, completed 35 Rapid Evidence Reviews, six Rapid Evidence Maps and 10 Rapid Evidence Summaries. We completed four primary research studies, with three published in peer reviewed journals, and seven ongoing. Our evidence informed policy decision-making and was cited in 19 Welsh Government papers. These included pandemic infection control measures, the Action Plan to tackle gender inequalities, and Education Renew and Reform policy. We conducted 24 Welsh Government evidence briefings and three public facing symposia.

**Policy implications:**

Strong engagement with stakeholder groups, a phased rapid evidence review approach, and primary research to address key gaps in current knowledge enabled high-quality efficient, evidence outputs to be delivered to help inform Welsh policy decision-making during the pandemic. We learn from these processes to continue to deliver evidence from March 2023 as the Health and Care Research Wales Evidence Centre, with a broader remit of health and social care, to help inform policy and practice decisions during the recovery phase and beyond.

**Supplementary Information:**

The online version contains supplementary material available at 10.1186/s41256-024-00354-1.

## Background

The COVID-19 pandemic showed the vital role research plays in informing policy and practice decisions to save lives [1]. Research was also needed to inform best strategies for managing direct and indirect harms of the pandemic, including increased surgical waiting lists and exacerbating inequalities [2–5]. However, traditional systematic literature reviews often take several years to complete and the academic journal publication process can be protracted with articles not always written in ways that make clear and practical recommendations. Sir Chris Whitty, the Chief Medical Officer for England during the pandemic, noted, ‘academics underestimate the speed of the policy process and publish excellent papers after a policy decision rather than good ones before it … the accurate synthesis of existing information is the most important offering by academics to the policy process [6].

The Wales COVID-19 Evidence Centre (WCEC), [7, 8] was established in March 2021 and funded for two years, to join the global effort to co-ordinate COVID-19 related research, and to address the lag between policy needs arising and available evidence [9]. We needed to: understand the impact of the pandemic on research priorities for the health and wider needs of people and communities in Wales; quickly and rigorously provide relevant evidence; and make this evidence available and actionable to our stakeholders involved in policy-making and delivery of health and social care. We describe the novel approaches we developed to meet these objectives. We also discuss our experiences, challenges and learning to inform our future vision as we transition to become the Health and Care Research Wales Evidence Centre from March 2023.

## Program implementation

**Fig. 1:**
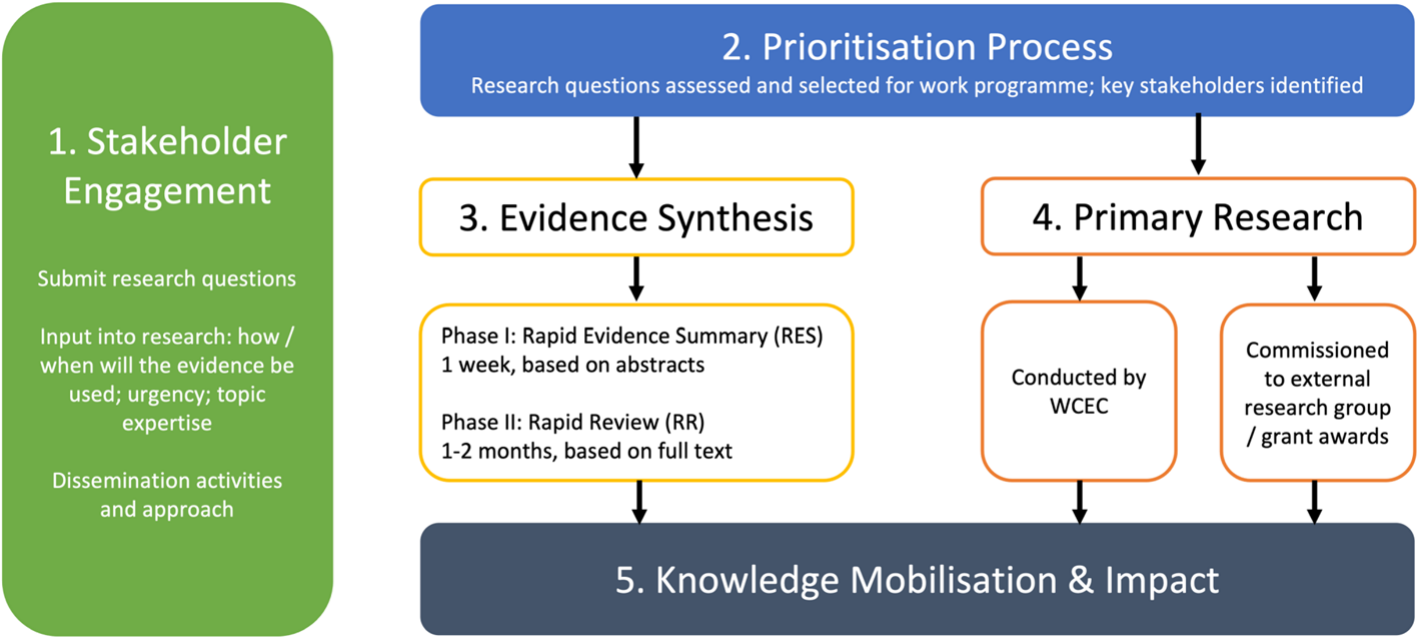
Wales COVID-19 Evidence Centre (WCEC) rapid evidence delivery processes

Our rapid evidence delivery processes are outlined in Fig. 1 and discussed below in more detail. These included: 1) stakeholder engagement throughout all processes [10, 11]; 2) research question identification and prioritisation [10]; 3) bespoke phased rapid evidence review methodology [12]; 4) rapid primary research [13–15]; and 5) knowledge mobilisation [16].

### Organisational structure

The WCEC operated with a core team and six Collaborating Partner research groups (Table 1). The core team included a Director (AE) and leads for, research identification and prioritisation and public involvement and engagement (NJW), stakeholder involvement (AC), rapid evidence synthesis (RL), rapid primary research methods (DW), and knowledge mobilisation (MG), with managerial support (AW, JG). During the pandemic, we worked closely with members of Welsh Government’s Technical Advisory Cell (RJL) who had a boundary spanning role to promote communication between the evidence centre and policy-makers; the role continues as the Welsh Government undergoes post-pandemic reorganisation.

**Table 1:** Wales COVID-19 Evidence Centre (WCEC) collaborating partner research groups

Abbreviation	Research Group	Research input	Website
BIHMR	Bangor Institute for Health and Medical Research, Bangor University	Rapid evidence synthesis	Welcome to the Bangor Institute for Health & Medical Research (BIHMR) | Health Sciences | Bangor University
HTW	Health Technology Wales, NHS Wales	Rapid evidence synthesis	Home—Health Technology Wales
PHWES	Public Health Wales Evidence Service, NHS Wales	Rapid evidence synthesis	Observatory—Public Health Wales (nhs.wales)
SURE	Specialist Unit for Review Evidence, Cardiff University	Rapid evidence synthesis	Specialist Unit for Review Evidence—Cardiff University
SAIL	Population Data Science, Swansea University	Analysis of anonymised linked data through the Welsh Secure Anonymised Information Linkage (SAIL) Databank	Population Data Science at Swansea University Medical School
WCEBC	Wales Centre for Evidence Based Care, Cardiff University	Rapid evidence synthesis	The Wales Centre For Evidence Based Care: A JBI Centre of Excellence—Research—Cardiff University

The core team worked closely with a Public Partnership Group, consisting of eight members [11]. Established in March 2022 following open recruitment through Health and Care Research Wales, these individuals represented the views of the public with regards to COVID-19 research and were involved in all stages of our evidence synthesis work. They wrote lay summaries to accompany our evidence reports and co-authored our publications (AS). Additional public partners were sought specifically (through open recruitment via Health and Care Research Wales) based on the primary research topic focus [11].

The six Collaborating Partner research groups are independent Welsh research teams based in Universities or the NHS, each with their own areas of domain and methodology expertise on which to draw, depending on the research question (Table 1). A fortnightly methodology subgroup meeting included representation from all Collaborating Partner review teams for shared learning and iteration of processes. We also liaised with other national and international research partners (e.g. International Public Policy Observatory, National Institute of Clinical Excellence (NICE), UK Health Security Agency) to avoid duplication of effort and ensure complementary analyses.

### Rapid evidence delivery process

#### Stakeholder engagement

Stakeholder engagement and collaboration was integral throughout our processes to ensure that we delivered research evidence that was timely, of the highest priority, and directly relevant to policy and practice [10]. Important COVID-19-related research questions were invited from various health and social care stakeholder groups during several rounds of the Stakeholder Research Question Prioritisation Exercise (ScoPE) process (described in Section 2 below). Key stakeholders were identified through an inclusive stakeholder mapping exercise and included the public, policy leads, health, education, and social care service delivery organisations and professionals [10, 11]. Further public engagement was sought via public facing symposium events in March 2021 and March 2022. We also conducted focus groups with communities that were disproportionately impacted by the pandemic to facilitate engagement, identify their priority questions and promote equity. This included black and ethnic minority groups, children and young people, housing association tenants, and disabled people [10, 11].

When a proposed question was adopted onto our work program, the relevant stakeholders (*n* = 2–3) and at least one public member were invited to join a series of online stakeholder meetings (usually three) with the lead Collaborating Partner research team to clarify the research question, identify the evidence need and the urgency, discuss early findings, contribute their expertise and knowledge of key articles / research, and become involved in dissemination of findings.

#### Research question identification and prioritisation

Our prioritisation process aimed to identify and select research questions that were of highest priority for COVID-19 focused health and social care policy and practice in Wales, [10, 11] in a situation where time did not allow for recognised formal prioritisation exercises such as James Lind [17]. Priority questions were invited through a bespoke, demand-driven Stakeholder Research Question Prioritisation Exercise (ScoPE) process via direct stakeholder consultation both within Welsh Government, and with external NHS, social care, professional, public, academic, industry and third sector groups [10]. The ScoPE process was formally conducted every six months, but was also reactive to accommodate emerging or urgent health, social care, or education research priorities needed to inform decision-making.

During the ScoPE exercise, stakeholder groups were invited to complete a proforma (please see supporting information 1) that ranked their ‘top research priorities’ (up to 10). Additional information requested included: relevance to the current or future COVID-19 context in Wales, importance of the evidence gap, potential benefits and for translation into practice, and urgency for the evidence. Submitted research questions were assessed against these criteria by the WCEC core team and public representatives for acceptance onto the work program. If necessary, further expert stakeholder advice was sought to clarify priorities and refine the research question. For efficiency, there was initial consideration of question overlap with work already undertaken or in progress (both within the Centre and externally), and whether evidence synthesis or primary research was needed. The work program was shared and discussed with Welsh Government representatives. Approved questions were then allocated to either the Evidence Synthesis Work Program or the Primary Research Work Program (see Sections 3 and 4 below).

#### Evidence synthesis work program

Research questions accepted onto the work program for evidence synthesis were allocated to one of our partner groups, with questions matched to experience within the group where possible. Our phased rapid review approach, [12] based on three types of products, was developed in line with international rapid review approaches to ensure we conducted and delivered robust, timely and efficient and effective evidence syntheses, [18–22] which also benefited from experience within the partner research groups [23–25].

##### Phase I: Rapid evidence summary (~ 1 week)

An initial introductory stakeholder meeting was set up, which included members from the WCEC core team and public representatives, the partner research group and key stakeholders. The meeting was held online and lasted about an hour. The aim was to clarify with the stakeholders the focus of the research question, how the evidence would be used, and proposed timelines.

The review team then conducted an exploratory review of key COVID-19 resources for existing reviews that may address the research question. A list of key resources was developed with information scientists to support the searches (see Supporting Information 2). This initial phase allowed the reviewers to familiarise themselves with the topic area, check the research question had not been addressed by another group and identify the likely extent and type of available evidence to inform the methods and design of the rapid review.

The output from this phase, based on abstracts and generally completed within a week, was presented as an annotated bibliography with key findings, called a *Rapid Evidence Summary (RES),* and discussed in a second online stakeholder meeting. This also provided the opportunity to present limited interim findings to stakeholders. If a relevant and current systematic review was identified that addressed the research question, then it could be summarised and appraised as a final product. For urgent decisions or where there was insufficient evidence to progress to a rapid review, the RES was published as the final product.

##### Phase II: Rapid review (1–2 months)

If sufficient evidence was identified in the RES, discussions during the second stakeholder meeting moved onto planning the *Rapid Review (RR).* This involved refining the research question and drafting the eligibility criteria (based on an evidence synthesis framework such as ‘PICO’) [26]. These discussions were also used to establish if there were particular *equality* considerations, and the potential *economic impact* of the evidence.

The rapid review was conducted using a variation of the systematic review approach, where components of the review process were abbreviated or omitted to generate the evidence to inform stakeholders within a short time frame whilst maintaining attention to bias. This offered the most rigorous and comprehensive product produced in a timely manner. As far as possible, the reviews followed methodological recommendations and minimum standards for conducting rapid reviews [18–22]. If timelines were tight, methodological decisions needed to be pragmatic. Approaches were described for transparency and included: a tertiary review (review of reviews), prioritising identified reviews for synthesis, and limiting searches for primary studies to countries with similar health and social care systems to the UK. When a focused review question could not be selected, an interim *Rapid Evidence Map (REM)* was conducted to support the selection of a substantive focus for the rapid review. The REM used abbreviated systematic mapping or scoping review methodology [27, 28]. The output from this phase was a rapid review report (template in Supporting Information 2) which was presented and discussed in a third online stakeholder meeting.

#### Primary research work program

**Fig. 2:**
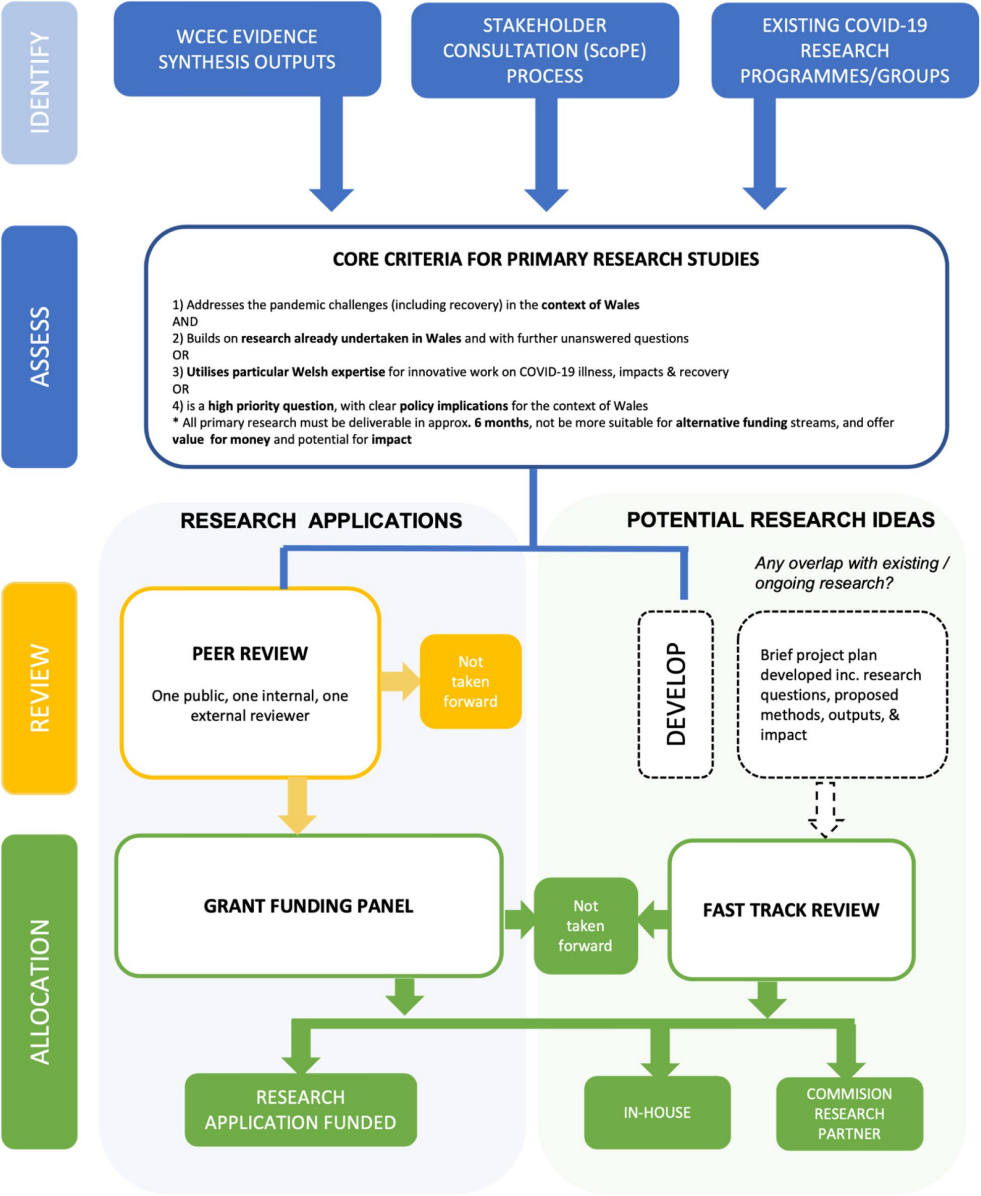
Wales COVID-19 Evidence Centre (WCEC) Primary Research Work Program topic identification, assessment, review, and allocation processes

This additional work program was set up in March 2022. Fig. 2 (an elaboration of process 4 in Fig. 1) outlines the topic identification, assessment, review and allocation processes, again designed to promote efficiency and effectiveness, and described below. Primary research projects were identified through three main routes: key gaps identified by WCEC evidence synthesis outputs; the ScoPE process (see Section 2) [10]; and though applications submitted by research groups. All questions identified via these three routes were subject to assessment against the ScoPE process described above and following criteria:

- Addressed the pandemic challenges (including recovery) in the context of Wales AND
- Built on research already undertaken in Wales and with further unanswered questions OR
- Utilised particular Welsh expertise for innovative work on COVID-19 illness, impacts & recovery OR
- Was a high priority question, with clear policy implications for the context of Wales.

All primary research needed to be deliverable in about 6 months, not be more suitable for alternative funding streams, and offer value for money and potential for impact. If criteria were met, researchers were invited to complete a full application form, which included further details regarding methods, pathway to impact and costings. Once received, the full application underwent peer review by one internal reviewer, one external reviewer (with topic / methodological expertise) and two public members. A grant funding panel met to discuss the applications, and successful projects received funding for the work.

We also had core team capacity and expertise to support a range of ‘in-house’ rapid primary research projects. Where the in-house team did not have the relevant specialist expertise, we commissioned appropriate Collaborating or external partners to conduct the work. For in-house and commissioned research, a similar process to the evidence synthesis program was used: three online meetings with the key stakeholders to clarify the research question, identify appropriate methods and analysis, provide expertise in the research process, and assist with knowledge mobilisation and the pathway to impact.

#### Knowledge mobilisation and impact

Our knowledge mobilisation processes were designed to ensure our products were accessible, timely and useful for our stakeholders to inform policy, practice and decision-making to promote effectivenss [16]. The process was iterative and tailored to meet the requirements of the stakeholders. The third online stakeholder meeting was used to present the findings from the evidence synthesis or primary research, address any queries, and support the development of a knowledge mobilisation plan, co-designed with the proposing stakeholders.

The templates for our rapid review final reports (Supporting Information 2) were based on recommendations for reporting evidence reviews for policy-makers and have been adapted for our primary research reporting [29]. For each report, a 'topline summary’ was developed highlighting the methodology, evidence base, research quality, key findings and implications for policy and practice. The report’s findings were also re-drafted into a lay summary by our public representatives to provide a widely accessible version, published alongside other outputs including infographics. Reports were published on pre-print servers and linked to the lay summaries on our WCEC website library.

Activities to promote the uptake and use of the evidence included fortnightly internal Welsh Government evidence briefings, where research findings were presented to a wider Welsh Government audience and invited key stakeholders. Here, implications of the evidence and practical next steps towards implementation were also discussed. A communication plan was in place, and we used social media through Health and Care Research Wales to disseminate findings including infographics and links to our review outputs and newsletters. We also recorded and tracked the impact of our work via ongoing engagement with stakeholders and an online survey. Our reports were made publicly available on our website, which was also linked to other COVID evidence resources (‘UK Health Security Agency- COVID Rapid Review Collections’ and internationally with COVID-END) [20, 30].

## Main achievements

### Stakeholder engagement

During the two years the centre was operational (March 2021–23), we reached out to 52 stakeholder groups across policy, health, social care, education, third sector and the public; 44 (85%) submitted COVID-19 related research questions [8, 10, 11]. During this time, 22 key stakeholders completed our survey to provide feedback on our processes and additional feedback was collected via meetings. Survey feedback showed that 21/22 (95%) were satisfied or very satisfied with our engagement processes, meetings and the final report; 100% trusted the report findings.

*‘The WCEC representatives involved with the project were highly responsive. Their suggestions were constructive, and they worked proactively to make meaningful progress that allowed our WG (Welsh Government) project team to quickly and conveniently locate relevant evidence that helped shape our policy proposals’.(WG Stakeholder)*

The eight members of the public in our Public Partnership Group were involved in a number of activities. These included: prioritising research questions for our work program, contributing to evidence reviews and engagement events, writing lay summaries and contributing to our newsletter and supporting the write-up of four publications for peer reviewed journals. They have also peer-reviewed research applications submitted for the primary research work program and attended Grant Funding Panels as panel members [11].

### Research Question identification and prioritisation

Three cycles of the ScoPE exercise were conducted (Spring 2021, Autumn 2021, Spring 2022). A total of 44 ScoPE forms were completed across the three cycles, with a total of 212 questions proposed via this route. We also received an additional 11 urgent Welsh Government requests [10]. Four focus groups were conducted with disproportionately affected groups between April and May 2022 to establish their ‘Top 10 research priorities’: children and young people (including representatives from the Centre for Development, Evaluation, Complexity and Implementation in Public Health Improvement’s ALPHA Group, Children’s Commissioner for Wales, Wolfson Centre), Disability Wales, Ethnic Minorities and Youth Support Team (EYST) Wales, and a local community housing group (Taff Housing, Cardiff) [11]. They proposed a total of 40 priority areas for research.

Attendees at the public facing WCEC symposium in March 2022 identified 57 priority areas (discussed during breakout rooms) which were ranked by participants following the event to establish their top 10 priorities to help inform our work program. After combining duplicate/similar themes/questions, a total of 58 questions were included in our work program [7, 8]. (This was reviewed every 3 months to ensure the questions still had relevance and clear pathways to impact.)

### Rapid evidence synthesis work program

Our evidence synthesis outputs conducted by our partner research groups (March 2021–23) included: 35 Rapid Reviews, six Rapid Evidence Maps and 10 Rapid Evidence Summaries [8]. Topics largely included education, inequalities, health and social care, for example, the effectiveness of innovations to address NHS surgical waiting lists and innovations to improve recruitment and retention of health and social care staff (Table 2).

*‘Excellent process for defining questions, setting boundaries for research, discussing key findings.’ (Stakeholder feedback on the review process)*

**Table 2:** Examples of rapid evidence synthesis products and impact

Research partner group	Date published	Study Title	Evidence Synthesis Product	Impact
Health Technology Wales	July 2021	Face coverings to reduce transmission of SARS-CoV-2	Rapid Review	To inform Welsh Government policy move to Alert level 0 (August 2021)
Specialist Unit for Review Evidence	July 2021	Direct harm from COVID-19 infection and COVID-19 vaccination in pregnant/post-partum women and the unborn child?	Rapid Evidence Summary	To support a Public Health Wales campaign to women and midwives (November 2021)
Public Health Wales	August 2021	The effectiveness of infection, prevention and control measures applied in education and childcare settings	Rapid Review	To inform Welsh schools’ re-opening (Sept 2021)
Wales Centre for Evidence Based Care	September 2021	The efficacy, effectiveness and safety of SARS-CoV-2 disinfectant methods (including ozone machines) in educational settings	Rapid Evidence Summary	To inform provision of CO2 monitors in Welsh schools (October 2021)
Wales Centre for Evidence Based Care	September 2021	Strategies to support learning and wellbeing among 16–19 yr old learners who have experienced significant disruption in their education as a result of the COVID-19 pandemic	Rapid Review	To inform the Welsh 16–19 Education Renew & Reform policy
Bangor Institute for Health and Medical Research	January 2022	Innovations to address inequalities experienced by women and girls during the COVID-19 pandemic	Rapid Review	To inform Welsh Action Plan to tackle gender inequalities (Jan 2022)
Biocomposites Centre, Bangor University	March 2022	The impact of COVID-19 induced changes in working practice on greenhouse gas emissions	Rapid Review	Inform Technical Advisory Cell advice (June 2022)

The WCEC core team also supported medical students at Cardiff University to conduct systematic reviews on: whether institutional racism contributed to adverse COVID-19 outcomes for ethnic minority healthcare staff; the effectiveness of antiracist interventions in healthcare; and the impact of the pandemic on homeless and prison populations. A living review exploring the risk of transmission of Sars-CoV-2 in vaccinated populations was conducted in collaboration with the UK Health Security Agency. An additional review team, The Biocomposites Centre, Bangor University, was commissioned to conduct a single review about the impact of the pandemic and changes in working practice on the environment, particularly greenhouse gas emissions. Several reviews over the pandemic highlighted evidence gaps and areas for further primary research, which were highlighted to funders.

### Primary research work program

Eleven studies were adopted onto our primary research work program (Table 3). Three grants were awarded to COVID-19 research teams; most studies were conducted ‘in-house’ or by our Collaborating Partner research teams. One study that required the research to be conducted through the medium of Welsh was commissioned to an external research group with appropriate expertise. Three of our completed primary studies were published in peer reviewed journals including the impact of the pandemic on cancer diagnosis in Wales, [13] and the diagnosis of 17 long term conditions including asthma, heart disease and diabetes [14]. Another cohort study showed that higher-risk adult community patients with COVID-19 in Wales treated with anti-viral therapy had a reduced the risk of hospitalisation or death [15]**.**

**Table 3:** Summary of studies adopted onto the WCEC primary research work program

Study Title	Lead	Timeframe
**Externally funded studies (grant awards)**		
Brain and Brainstem Bases of Long COVID (BBB COV)	Cardiff and Vale University Health Board and Cardiff University Brain Research Imaging Centre	Feb 22 – Mar 23
Public views during the coronavirus pandemic (PVCOVID)	Swansea University	Jun 22 – Mar 23
COVID-19 and common respiratory tract infection-related health behaviours: development of community-based approaches to reducing the burden of RTIs in Wales	Swansea University	Sept 22 – Mar 23
**In-House and Collaborating Partner (WCEC Team) studies**		
Impact of the SARS-CoV-2 pandemic on female breast, colorectal and non-small cell lung cancer incidence, stage and healthcare pathway to diagnosis during 2020 in Wales	SAIL Databank	Complete and published Br J Cancer 2022 [13]
Impact of the COVID-19 pandemic on diagnosis for patients with long term conditions	SAIL Databank	Complete, paper published BJGP[14]
Monoclonal Antibody and Antiviral Evaluation	SAIL Databank	Complete, paper published Journal of Infection (15)
Evaluating equality in COVID-19 vaccine uptake	Public Health Wales Observatory and SAIL Databank	Jul 22 – Mar 23
Survey to explore the experiences of using carbon dioxide monitors to improve ventilation in education settings in Wales	WCEC Team	Complete Sept 22
Critical review and refinement of a PRIME patient safety tool to explore patient and public safety concerns in Wales in relation nosocomial COVID-19 infections	WCEC Team	Sep 22 – Mar 23
The mental health and wellbeing of health and social care workers (HSCWs) during the recovery phase of the COVID-19 pandemic within Wales, United Kingdom: establishing a longitudinal survey	WCEC Team	Aug 22 – Mar 23
**Commissioned studies**		
Looking to the future post-covid: exploring the perceptions and experiences of learners in Welsh-medium education and families where Welsh is not spoken	Commissioned to Bangor and Aberystwyth Universities (and WCEC Team)	Aug 22 – Mar 23

### Knowledge mobilisation and impact

Our work program, 53 reports (including 26 lay summaries in English and Welsh written by public members) and three Newsletters were published as open access via our website library [7, 8]. We conducted 24 fortnightly Welsh Government evidence briefings (usual attendance 20–30 people) and findings were also presented to wider groups. For example, our healthcare education report was presented to UK heads of medical education; Long-Covid work was presented to the Senedd cross-party on Long-COVID; and the vaccination in pregnancy findings were presented to the heads of maternity in Wales. We held three public facing symposia (70–80 attendees) to help disseminate outputs and generate discussion on impacts and evidence gaps opened with presentations by senior Welsh Government Ministers or policy officials. The symposia presented findings from themes of work including: education and young people (Dec 2021), impact of the evidence centre (Mar 2022), and inequalities and vulnerable gorups (Sept 2022) [7, 8, 16].

*‘The Symposium was very encouraging… in bringing academics and Welsh Government together to produce evidence-based policy and practice… also pleasing to hear that patient and public experience is at the heart of the Centre.’ Chair of the SUPER Group, public contributors who support research activities at PRIME Centre Wales*

Our reports informed policy decision-making, with 19 of our reports referenced in Welsh Government papers (Table 2). Examples include: rapid reviews of face coverings to inform the move to Alert level 0 (August 2021); infection control measures in schools to inform schools’ re-opening in Sept 2021; disinfection methods in schools regarding ozone/CO_2_ monitors in October 2021; vaccination for pregnant women (Public Health Wales campaign to women and midwives, Nov 2021); the Action Plan to tackle gender inequalities (Jan 2022); impact of COVID-19 on greenhouse gas emissions (June 2022); and 16–19 Education into Renew & Reform policy. One review directly led to re-profiling of £3 million funding towards provision of CO_2_ monitors for schools across Wales [31].

In addition to publication on our website library, final reports were published on pre-print servers including medRxiv. This enabled wider sharing and the collection of metrics. For example, the abstract of a rapid review on the effectiveness of interventions and innovations relevant to the Welsh NHS context to support recruitment and retention of clinical staff, [32] was viewed 671 times, and the pdf was downloaded 181 times in the first 3 months. While some evidence was not directly included in Welsh Government papers, feedback from stakeholders indicated that information confirmed their knowledge and was useful:

*‘The rapid evidence review highlighted that a multi-strategy approach was required. This reinforced the approach that was being taken and ensured that our practice was supported by the evidence available at that time.’ (Stakeholder feedback)*

## Policy implications

### Key outputs

In two years of operation (March 2021–23), the WCEC engaged with 44 stakeholder groups across policy, health, social care, education, third sector and the public. Evidence synthesis outputs included: 35 Rapid Reviews, six Rapid Evidence Maps and 10 Rapid Evidence Summaries [8]. We completed four primary research studies, three published in peer-reviewed journals, and seven ongoing [13–15]. Our evidence informed policy decision-making and was cited in 19 Welsh Government papers. We conducted 24 Welsh Government evidence briefings, three public facing symposia and received positive feedback from stakeholders on our review process and timely accessible products [8, 16].

### Strengths and limitations

Our strengths include setting up the evidence centre and collaboration with the Welsh research groups in a short timeframe to assist the pandemic effort; also strong stakeholder engagement to ensure the correct questions were being asked and the evidence contributed to decisions. Our phased rapid review approach to identify existing work and publication of our work program on our website for global transparency avoided duplication of effort with other research groups, a problem recognised during the pandemic [33]. Incorporating the primary research work program to address evidence gaps identified from the evidence synthesis work is novel. Our demand driven approach used rapid methods to deliver evidence to meet the needs and timeframes of our stakeholder decision-makers. We developed various products to distil the evidence and improve impact. We recruited a public group because we can only ‘learn to live’ with COVID by learning from those who have lived through it. We encouraged student involvement, giving them an opportunity to learn about our processes and take on questions that require a longer timeframe with more rigorous methods (systematic reviews). We actively engaged with other research groups to avoid duplication and encouraged collaboration across different NHS, policy and academia groups.

Our short timeframes were a limitation, requiring modification to the systematic review process, such as one reviewer extracting data or conducting a quality assessment. Risks of error were mitigated through strong stakeholder engagement—identifying key papers and discussing and querying findings, also being transparent about the limitations of the analyses and syntheses. Challenges included meeting stakeholder expectation about what evidence can be delivered in a short time period and understanding limitations and quality of the available evidence. We mitigated this through discussion in the stakeholder meetings and ongoing email communication. Setting up online stakeholder meetings with representatives from all groups in short time frames with different timescales to traditional research projects was also a challenge, email communication was again used if stakeholders were unable to make a meeting. Specific primary research challenges included timely ethical approval, participant recruitment and ensuring Welsh language requirements were met. We plan to publish papers on each of the individual processes (prioritisation, rapid reviews, rapid primary synthesis and knowledge mobilisation) to describe this learning at each stage in more detail [10–12, 16].

### Comparison with other approaches

The COVID-19 pandemic highlighted the need for research evidence and its value in informing policy decisions, with the mantra of ‘Following the Science’ becoming common place. However, scientists may find themselves crossing boundaries and taking on public figure roles to which they are unaccustomed [34]. Pielke describes four idealised roles for scientists in decision-making: the *pure scientist* with no connection with how the evidence is understood and interpreted by Government Officials; the *issue advocate* who focusses on the implications of research for a particular political agenda; the *science arbiter* that responds to specific scientific questions raised by decision-makers; and the *honest broker* who seeks to integrate scientific knowledge with stakeholder concerns in the form of alternative possible courses of action [35]. Our rapid evidence reviews reflect the science arbiter role, focusing on a narrow research question to enable the review to be completed in a timely manner. However, by highlighting evidence gaps and addressing some of these in our primary research work program, the centre may be considered as an honest broker role—providing research relevant to the Welsh context and evidence for alternative options.

How the evidence is understood and interpreted by Government Officials, including politicians and policy-makers, and service leads, is also a challenge. Research-policy engagement initiatives can take many forms, most aiming to improve research dissemination or create relationships, but often not evaluated [36–38]. We have used strategies such as communication with stakeholders to tailor the research accordingly and short, concise and freely available reports in plain language to help at this interface. However, there is currently a lack of evidence regarding how to enhance the “evidence literacy” of policy decision-makers and “policy literacy” of scientists to enable a culture or environment to facilitate their collaboration and deliver both timely policy-driven evidence synthesis and evidence-based policy-making [39]. Our learning includes: engaging with stakeholders and public partners that will use the evidence; delivering an evidence product in time to help with decision-making; being clear about data quality and potential limitations of the evidence; sharing work programs to avoid duplication of effort with other research groups; and planning knowledge mobilisation strategies and the most useful outputs with stakeholders from the start [8]. These lessons learned are helpful as we transition to the Health and Care Research Wales Evidence Centre (March 2023), [40] and may be transferable to other evidence centres.

### Further research

Further research is needed to understand and evaluate how to facilitate knowledge transfer at the Science-Policy-Practice-Interface and to evaluate the long term effectiveness and impact of evidence centres or similar units.

## Conclusions

Strong engagement with stakeholder groups, a phased rapid evidence review approach, and primary research to address key gaps in current knowledge have enabled high-quality, efficient evidence outputs to be delivered, to effectively help inform Welsh policy decision-making during the pandemic. We learn from these processes to continue to deliver evidence to help inform policy and practice decisions in the post-pandemic recovery phase as the Health and Care Research Wales Evidence Centre.

### Supplementary Information

**Supplementary Material 1.**

## Data Availability

Not applicable.

## Abbreviations

BIHMR: Bangor Institute for Health and Medical Research
HCRW: Health and Care Research Wales
HTW: Health Technology Wales
KM: Knowledge Mobilisation
PHWES: Public Health Wales Evidence Service
PPG: Public Partnership Group
RES: Rapid Evidence Summary
REM: Rapid Evidence Map
RR: Rapid Review
SAIL: Welsh Secure Anonymised Information Linkage Databank
SPPI: Science Policy Practice Interface
ScoPE: Stakeholder Research Question Prioritisation Exercise
SURE: Specialist Unit for Review Evidence
TAC: Welsh Government’s Technical Advisory Cell
WCEC: Wales COVID-19 Evidence Centre
WCEBC: Wales Centre for Evidence Based Care
