# Supplementary material for: Informing evidence-based policy during the COVID-19 pandemic and recovery period: learning from a national evidence centre
Source: Glob Health Res Policy. 2024 May 31;9:18. doi: 10.1186/s41256-024-00354-1 (PMC11140997; doi:10.1186/s41256-024-00354-1)
Supplement: Supplementary file 1 — Supplementary Material 1. [file 41256_2024_354_MOESM1_ESM.pdf]

Supporting Information 1: ScoPE proforma

COVID-19 Evidence Centre - Stakeholder Research Question Prioritisation Exercise (ScoPE) Proforma

PART A		
**Stakeholder Information:** ***tell us about your organisation or group***		
**Stakeholder Name**<br>*Name & geographical location(s) of your organisation / group*	**Stakeholder Role**<br>*Describe the main role your organisation / group plays in the health and care of people in Wales*	**Sector**<br>*Which sector best describes your organisation? (tick all that apply)*
**Your Information:** ***tell us about your role in the organisation or group***		
**Contact Name**<br>*Name, title*	**Your Role**<br>*Tell us about your main role within the organisation / group*	**Contact details**<br>*Email, telephone*

<table>
<tr><th colspan="2">PART B</th></tr>
<tr><td colspan="2">Stakeholder Research Question Prioritisation Exercise (ScoPE) Process: tell us about the prioritisation process in your organisation or group</td></tr>
<tr><td>Who have you involved in this exercise?
e.g. names of key individuals, members, wider groups / organisations consulted</td><td>How many people contributed to this exercise?</td></tr>
<tr><td rowspan="3"></td><td></td></tr>
<tr><td>When was the exercise completed? e.g. March 2021</td></tr>
<tr><td></td></tr>
<tr><td colspan="2">About your research question prioritisation exercise (ScoPE) process
Tell us how you have gone about identifying key research questions for your organisation / group.
Describe how you consulted the individuals and groups listed above and how you came to agreement on the questions you have put forward.</td></tr>
<tr><td colspan="2"></td></tr>
</table>

PART C						
**Top Research Questions** - ***provide up to 10 research questions and rank these questions in order of greatest need and potential for impact***						
**Your Research Question(s)**<br>*Please see guidance on making questions specific*		**Relevance to the current or future COVID-19 context in Wales**	**Importance of the evidence gap - what is the scale, cost & consequence of the issue?**	**What would be the potential benefits that could result from a summary of best evidence?**	**How would you use this evidence to achieve the benefits described?**	**When would you need this information for it to be helpful to you?**
**1**						
**2**						
**3**						
**4**						
**5**						
**6**						
**7**						

PART D
Future Priorities
The impact of the pandemic on health and care in Wales is continually changing. Questions that have been identified may become more or less important depending on the phase of the pandemic. In addition, new issues will emerge and become more important.<br><br>In order to be proactive to longer-term evidence needs, we are asking stakeholder groups to think about potential future priorities as we move through subsequent phases of the pandemic.<br><br>Please list potential future priorities below, giving an indication of timeframe (e.g. Winter 2021/2022; return of schools / Universities for 2021/2022 academic year), and briefly explain why you think these will be important.

Thank you for completing the Stakeholder Research Question Prioritisation Exercise (ScoPE)

Please return completed ScoPE Proformas via email to:

WC19EC@cardiff.ac.uk

Supporting Information 2: Rapid review template and COVID resources

Wales COVID-19 Evidence Centre
Canolfan Dystiolaeth COVID-19 Cymru

Ariennir gan Lywodraeth Cymru
Funded by Welsh Government

Ymchwil Iechyd a Gofal Cymru
Health and Care Research Wales

Wales COVID-19 Evidence Centre (WCEC)

Rapid Review

[Insert title of rapid review]

Report number – RRXXX *[the report number will be added by the WCEC]* (Insert Month & Year)

**Rapid Review Details**
**Review conducted by:**<br>[Enter Collaborating Partner Name]<br><br>**Review Team:**<br>▪ [Enter Collaborating Partner's team members names]<br>▪ [Enter Collaborating Partner's team members names]<br><br>**Review submitted to the WCEC on:**<br>[day, month, year]<br><br>**Stakeholder consultation meeting:**<br>[day, month, year]<br><br>**Rapid Review report issued by the WCEC on:**<br>[day, month, year]<br><br>**WCEC Team:**<br>▪ [Enter team names involved in drafting, Topline Summary, editing etc]<br>▪ [Enter team names involved in drafting, Topline Summary, editing etc]<br><br>**This review should be cited as:**<br>[WCEC team to insert citation – once agreed, with hyperlink]

Disclaimer: The views expressed in this publication are those of the authors, not necessarily Health and Care Research Wales. The WCEC and authors of this work declare that they have no conflict of interest.

PLEASE NOTE: This section will be added by the WCEC upon receipt of the RR.

[Insert title of rapid review]

Report number – RRXXX (Insert Month & Year)

TOPLINE SUMMARY

What is a Rapid Review?

Our rapid reviews (RR) use a variation of the systematic review approach, abbreviating or omitting some components to generate the evidence to inform stakeholders promptly whilst maintaining attention to bias. They follow the methodological recommendations and minimum standards for conducting and reporting rapid reviews, including a structured protocol, systematic search, screening, data extraction, critical appraisal, and evidence synthesis to answer a specific question and identify key research gaps. They take 1- 2 months, depending on the breadth and complexity of the research topic/ question(s), extent of the evidence base, and type of analysis required for synthesis.

Who is this summary for?

[summary of intended audience; potential for use]

Background / Aim of Rapid Review

[Brief summary of the background / aim / research question – 1-2 sentences]

Key Findings

[xxxx studies were identified]

Extent of the evidence base

- [insert key finding] insert as many as required
- [insert key finding]

Recency of the evidence base

- [provide an indication of when the searches were conducted, e.g. 'The review included evidence available up until November 2022', then if needed, also provide a summary of the recency of the included evidence.]

Evidence of effectiveness

- [insert key finding]
-

Best quality evidence

- [insert key finding]
- [insert key finding]

Policy Implications

- [insert policy implications] insert as many as required
- [insert policy implications]
- [insert policy implications]

**Strength of Evidence**
[summarise this]

TABLE OF CONTENTS

TABLE OF CONTENTS 9
1. BACKGROUND 11
  1.1 Who is this review for? 11
  1.2 Purpose of this review 11
2. RESULTS 11
  2.1 Overview of the Evidence Base 11
  2.2 Effectiveness of XXX for XXX 11
    2.2.1 [Add relevant sub headers as required] 12
    2.2.3 XXX 12
    2.2.2 Bottom line results for XX 12
  2.3 Effectiveness of XXX for XXX 14
    2.3.1 14
3. DISCUSSION 15
  3.1 Summary of the findings 15
  3.2 Strengths and limitations of the available evidence 15
  3.3 Implications for policy and practice 15
  3.4 Strengths and limitations of this Rapid Review 15
4. REFERENCES 16
5. RAPID REVIEW METHODS 17
  5.1 Eligibility criteria 17
  5.2 Literature search 17
  5.3 Study selection process 18
  5.4 Data extraction 18
  5.7 Quality appraisal 18
  5.8 Synthesis 18
6. EVIDENCE 19
  6.1 Study selection flow chart 19
  6.3 Data extraction tables 19
  6.3 Quality appraisal tables 19
  6.2 Information available on request 19
7. ADDITIONAL INFORMATION 20
  7.1 Conflicts of interest 20
  7.2 Acknowledgements 20
8. ABOUT THE WALES COVID-19 EVIDENCE CENTRE (WCEC) 21

9. APPENDIX 22

Abbreviations:

Acronym	Full Description
	[Add key abbreviations]

1. BACKGROUND

1.1 Who is this review for?

This Rapid Review was conducted as part of the Wales COVID-19 Evidence Centre Work Programme. The above question was suggested by XXXX [Report the Stakeholder Group that submitted the review question (WCEC core team may have this information when unclear); do not report individual names. Provide a summary of intended audience and intended use of the evidence]

1.2 Background and purpose of this review

[Where possible, do not copy tables or text from other sources/software, as this makes editing difficult if it's not Word]

[Provide **high-level** description of the background / context / why this review is important

Include clear statement of the research question at the end of this section]

2. RESULTS

2.1 Overview of the Evidence Base

[Describe the extent and overall nature of evidence base here along with an indication of the structure of the results section and location of further details or included studies / Tables.

Wherever possible, report the results grouped by outcome and/or comparison or by relevant subgroups/themes. Provide a synthesis of the findings, rather than list the results of each individual study separately.

Using relevant sub-headers provide a summary of the evidence, including nature, key findings, and strength of the evidence and evidence gaps for each question and or relevant sub-category.]

2.2 Effectiveness of XXX for XXX

[Amend the title according to the review question]

Do not use numbered headers greater three levels (i.e. 3.2.1.); where necessary use different text/styles for additional (non-numbered) sub-headers. Unless that is, further numbered sub-headers are specifically required for clarity (e.g. when describing a complex evidence base).

2.2.1 [Add relevant sub headers as required]

[Summarise the evidence according to relevant subsections, comparison, or important subgroups, which may be dependent on the review question or emerge during data extraction]

2.2.2 XXX

2.2.3 Bottom line results for XX

[Include a bottom-line summary for each subsection]

Table 1: Summary of …

[The type of table included here will vary depending on the review question and study design. The data extraction needs to be succinct. However, if these are lengthy, consider including after the narrative summary, or under Included studies (section 6) with an abbreviated table here providing an overview of the evidence base or overall findings].

Example of table for secondary research

Citation (Country)	Review details	Included studies	Quality	Findings and observations/notes
**Autor, year (Country)**	**Review period:**<br><br>**Review purpose:**<br><br>**Included study designs:**<br><br>**Included outcome measures:**	**Number of included studies:**<br><br>**Key characteristics:**<br>[e.g. date, study designs, number and type of participants, countries – descriptors (and sub-headers) will depend on what's relevant to the review question;<br><br>If feasible. consider listing included studies to identify overlap]		

Abbreviations:

Example of table for primary studies

Citation (Country)	Study Details	Participants & setting	Key findings	Observations/notes
**Autor, year (Country)**	**Study Design:**<br><br>**Type of intervention [*exposure*]:**<br><br>**Data collection methods:**<br><br>**Quality rating:**	[Structure will depend on study design; may not need sub-headers]<br><br>**Sample size:**<br><br>**Participants:**<br><br>**Setting:**<br><br>**Dates of data collection:**	[Structure will depend on review question]<br><br>**Primary Findings:**<br><br>**Additional Findings:**	

Abbreviations:

2.3 Effectiveness of XXX for XXX

[Add separate headers for any additional review questions]

2.3.1

2.2.3 XXX

2.2.2 Bottom line results for XX

[Include a bottom-line summary for each subsection]

3. DISCUSSION

3.1 Summary of the findings

[Add further sub heads as required]

3.2 Strengths and limitations of the available evidence

[this incorporates areas of uncertainty]

3.3 Implications for policy and practice

Add implications for future research where necessary]

3.4 Strengths and limitations of this Rapid Review

4. REFERENCES

[insert references Harvard format as per the house style]

5. RAPID REVIEW METHODS

5.1 Eligibility criteria

[Report the eligibility criteria according to the Participant, Intervention (exposure), Comparison, Outcomes (PICO) framework **or alternative** appropriate framework, e.g. Population, Phenomenon of Interest, Context (PICo). Multiple questions will require separate eligibility criteria.

This information can be presented as structed text or using a table format. Clarify where indirect evidence, e.g. from other pandemics or pre-covid are considered]

Example of a table format – **amend headers as necessary**, depending on relevancy and Question type.

Table X: Eligibility criteria

	**Inclusion criteria**	**Exclusion criteria**
**Participants**		
**Settings**		
**Intervention / exposure**		
**Comparison**		
**Outcomes**		
**Study design**		
**Countries**		
**Language of publication**		
**Publication date**		
**Publication type**	Published and preprint	

[**Provide Key definitions where necessary**]

5.2 Literature search

[Report when the searches were carried out, the sources searched, and search terms or search strategy used. Report any search limits used]

5.3 Study selection process

[Report how studies were selected for the review, including the number of reviewers involved and how discrepancies were resolved.]

5.4 Data extraction

List the data extracted, the number of researchers involved and how accuracy was assured

5.7 Quality appraisal

5.8 Synthesis

5.9 Assessment of body of evidence

6. EVIDENCE

6.1 Study selection flow chart

6.3 Data extraction tables

6.3 Quality appraisal tables

[Provide summary tables here (templates are available); not sperate forms for individual studies]

6.2 Information available on request

[Examples include protocol, search strategies, lengthy data extraction tables, excluded studies. Additional information such as search strategy for MEDLINE and quality appraisal tables can be added as an Appendix]

7. ADDITIONAL INFORMATION

7.1 Conflicts of interest

[Example text: The authors declare they have no conflicts of interest to report.]

7.2 Acknowledgements

[Example text: The authors would like to thank XX, XX, XX, and XX for their contributions during stakeholder meetings in guiding the focus of the review and interpretation of findings.]

8. ABOUT THE WALES COVID-19 EVIDENCE CENTRE (WCEC)

The WCEC integrates with worldwide efforts to synthesise and mobilise knowledge from research.

We operate with a core team as part of Health and Care Research Wales, are hosted in the Wales Centre for Primary and Emergency Care Research (PRIME), and are led by Professor Adrian Edwards of Cardiff University.

The core team of the centre works closely with collaborating partners in Health Technology Wales, Wales Centre for Evidence-Based Care, Specialist Unit for Review Evidence centre, SAIL Databank, Bangor Institute for Health & Medical Research/ Health and Care Economics Cymru, and the Public Health Wales Observatory.

Together we aim to provide around 50 reviews per year, answering the priority questions for policy and practice in Wales as we meet the demands of the pandemic and its impacts.

Director:

Professor Adrian Edwards

Contact Email:

WC19EC@cardiff.ac.uk

Website:
https://healthandcareresearchwales.org/about-research-community/wales-covid-19-evidence-centre

9. APPENDIX

Resources searched during Rapid Review Searching

[A single list of resources has been developed for guiding and documenting the sources searched as part of a Rapid Review (see separate document). All 'core' resources should be searched, but other resources may be considered if appropriate to the topic, or time allows.

List the resources used and record the search strategies used below the table.]

Table X: Resources searched [Delete and add resources to this table as required]

Resource	Number of hits
Core COVID-19 specific resources	
Cochrane COVID Review Bank<br>https://covidreviews.cochrane.org/search/site	
WHO Global Coronavirus Database<br>https://search.bvsalud.org/global-literature-on-novel-coronavirus-2019-ncov/	
L*OVE COVID<br>https://app.iloveevidence.com/loves/5e6fdb9669c00e4ac072701d?utm=aile	
Cochrane COVID-19 Study Register<br>https://covid-19.cochrane.org/	
VA-ESP<br>https://www.covid19reviews.org/index.cfm	
Core non-COVID-19 specific resources	
Medline/PubMed	
Embase	
Cochrane Library	
Ongoing clinical trials	
clinicaltrials.gov<br>http://clinicaltrials.gov/	
WHO ICTRP<br>http://apps.who.int/trialsearch/	
Additional COVID-19 resources	
Trip – for guidelines	
COVID-END<br>https://www.mcmasterforum.org/networks/covid-end	
COVID-19 Evidence Alerts from McMaster PLUS™<br>https://plus.mcmaster.ca/COVID-19/	
Secondary resources for reviews relevant to local/UK context	
United Kingdom Health Security Agency (UKHSA) – COVID-19 Rapid Reviews<br>https://ukhsalibrary.koha-ptfs.co.uk/covid19rapidreviews/	
NICE resources for COVID reviews<br>https://www.nice.org.uk/guidance/conditions-and-diseases/respiratory-conditions/covid19/products?Status=Published	
Healthcare Improvement Scotland – COVID-19: Evidence for Scotland<br>http://www.healthcareimprovementscotland.org/our_work/coronavirus_covid-19/evidence_for_scotland.aspx	

Ireland, HSE Library, COVID-19 Summaries of Evidence<br>https://hselibrary.ie/covid19-evidence-summaries/	
HIQA Health Information and Quality Authority (Ireland) – Rapid reviews<br>https://www.hiqa.ie/reports-and-publications/health-technology-assessments?tid_1=All&field_hta_topics_target_id=112	
Secondary resources for reviews produced by key international organisations	
NCCMT COVID-19 rapid reviews (Canada)<br>https://www.nccmt.ca/covid-19/covid-19-rapid-evidence-service	
ECDC European Centre for Disease Prevention and Control (COVID-19 outputs)<br>https://www.ecdc.europa.eu/en/publications-data	
CDC centre for Disease Control and Prevention - Guidance for COVID-19 (US)<br>https://www.cdc.gov/coronavirus/2019-ncov/communication/guidance.html	
AHRQ Agency for Healthcare Research and Quality (US)<br>https://www.ahrq.gov/coronavirus/health-systems-research.html	
NASEM The National Academy of Sciences Engineering Medicine - Coronavirus Resources Collection (US)<br>https://www.nap.edu/collection/94/coronavirus-resources	
Australian National COVID-19 Clinical Evidence Task Force - Living Guidelines<br>https://covid19evidence.net.au/	
Secondary research resources for (non-COVID-19) reviews	
Trip – for guidelines	
Campbell Collaboration	
JBI (via OVID)	
Epistemonikos	
International HTA database (INAHTA-HTA)	
PROSPERO	
Additional resources searched	
Google Advanced Search	
Google Scholar	
